# A Course of Lectures on Dental Physiology and Surgery

**Published:** 1849-04

**Authors:** 


### Bibliographical Notices.

*A course of Lectures on Dental Physiology and Surgery,* delivered at the Middlesex Hospital School of Medicine, by JOHN TOMES, surgeon dentist to the Middlesex Hospital. London: JOHN W. PARKER, 1848, pp. 392, 8vo.

With the lectures which form the above named work, our readers are already familiar, they having been published in the Journal, but in bringing them out in book form, the author has subjected them to a thorough revision, and illustrated them with new, and more than double the number of engravings which accompanied their first publication. The work, as it now appears, constitutes a valuable accession to the literature of dental science. Mr. Tomes is a good writer, and is evidently familiar with the writings of most of the best authors on the teeth, and especially, those upon the anatomy and physiology, and microscopical structure of these organs.

The first six lectures, as our readers will recollect, are devoted to the anatomy and physiology of the teeth, embodying a condensed summary of the recent researches into their microscopical structure. But it is not necessary, as all the lectures have already been published in the Journal, to enter into a critical analysis of the work, our object in noticing it at this time, being merely to acquaint our readers with the fact of the publication of the lectures in book form. We might, were we disposed, though approving most of the opinions and doctrines of the author, point out some, which we conceive to be evidently erroneous, bût as we are so well pleased with the work as a whole, we will not permit ourself, at this time, to indulge in fault finding. We may, however, be permitted to say, in conclusion, that as the lectures were delivered to medical students, the author has entered but little into practical detail. He has confined himself more particularly to dental physiology and pathology, than to practical dentistry, and in these departments he has acquitted himself in a very able and creditable manner. It is a work, therefore, which should be in the library of every dentist.

